# Draft genome sequence of biofilm-forming *Pseudomonas aeruginosa* HLHR and non-biofilm-producing *Pseudomonas* sp. HLMP isolated from vermicompost

**DOI:** 10.1128/mra.00002-25

**Published:** 2025-06-03

**Authors:** Niteesh Kumar Pandey, Alaa Eddin Alhmeidi Alkhatib, Saugata Hazra

**Affiliations:** 1 Department of Bioscience and Bioengineering, Indian Institute of Technology-Roorkee 30112, Roorkee, Uttarakhand, India; 2 Centre for Nanotechnology, Indian Institute of Technology-Roorkee 30112, Roorkee, Uttarakhand, India; The University of Arizona, Tucson, Arizona, USA

**Keywords:** whole genome, *Pseudomonas aeruginosa*, vermicompost

## Abstract

We announce the draft genomes of *Pseudomonas aeruginosa* HLHR and *Pseudomonas* sp. HLMP isolated from a vermicompost sample.

## ANNOUNCEMENT

*Pseudomonas* are gram-negative aerobic bacteria commonly present in most environments. Members of this genus, such as *Pseudomonas aeruginosa*, can form biofilms and cause several nosocomial infections where biofilms contribute to antibiotic resistance in humans (1).

*Pseudomonas* sp. HLMP and *Pseudomonas aeruginosa* HLHR were isolated from ready-to-use fully matured vermicompost, collected in March 2021 from Mandsaur (24.1027979 N 75.1108802 E), Madhya Pradesh, and Farukhnagar (28.4489896 N 76.8227916 E), Haryana, India, respectively. To estimate the bacterial load, the samples were serially diluted in 0.9% saline solution, plated on *Pseudomonas* Isolation Agar Base (HiMedia), and incubated at 37°C for 24 hours (2, 3). A single colony was picked and grown in LB Broth at 37°C for 24 hours (HiMedia). Silica membrane-based automated QIAamp 96 DNA QIAcube HT Kit (Qiagen) was utilized for genomic DNA isolation. The TruSeq Nano DNA Library Kit (Illumina) was used for WGS library preparation (4). The Illumina Hi-Seq 2000 platform was used for sequencing.

**Fig 1:**
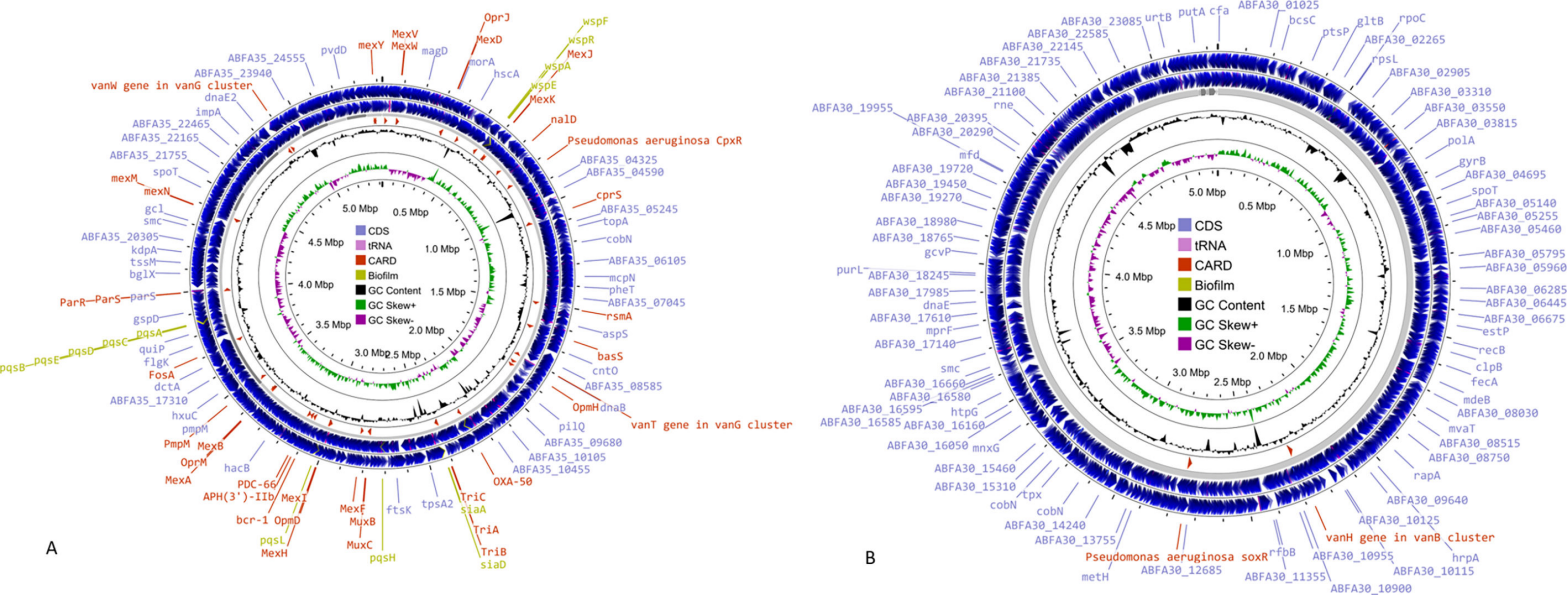
The genome maps of the draft genome were generated using Proksee (https://proksee.ca/, accessed on 27 December 2024). Coding DNA sequences (CDSs) are represented by blue arrows, CARD-RGI genes are highlighted with red arrows, and biofilm biosynthetic genes are denoted by light green color. GC content, GC skew−, and GC skew+ are represented by black, purple, and green peaks, respectively. (**A**) Genomic map for *Pseudomonas aeruginosa* HLHR. (**B**) Genomic map for *Pseudomonas* sp. HLMP.

During sequencing, 9.693150 (HLHR) and 7.814916 (HLMP) million raw reads (forward + reverse), with a read length of 151 bp, were obtained. By using the fastp 0.20.0 tool (5), quality filtering was performed, resulting in a total of 6.919896 (HLMP) and 8.666272 (HLHR) million high-quality (HQ) reads (forward and reverse). The HQ reads had a length of 139 bp for HLMP and 138 bp for HLHR. SPAdes 1.0.4 sequence assembler was used for the primary assembly (6) (Table 1) and provided contigs with minimum and maximum sequence lengths of 206 and 560,447 bp for HLHR, while 295 and 654,600 bp for HLMP. The location and size of HLHR (1,530 bp) and HLMP (1,531 bp) 16s rRNA genes in the genome were predicted by the barrnap 0.9 tool (7). NCBI-BLAST (blastn) search HLHR identified 99.90% identity with *Pseudomonas aeruginosa* (Accession_ID NR_026078.1)*,* while HLMP showed 99.98% with *Pseudomonas stutzeri* (also known as *Stutzerimonas stutzeri*) (Accession_ID NR_103934.2) as the closest genome. Read alignment was done by using the aligner Bowtie2 (version 2.3.4.1), where the assembled genome fasta file was used as reference, and filtered HQ reads of all samples were aligned to the respective assembled genome (8). Gene annotation was performed by the NCBI Prokaryotic Genome Annotation Pipeline version 6.7 (Table 1) (9). WGS analysis of the HLHR strain reveals multiple biofilm biosynthesis genes such as wsp (A, E, F, and R), pqs (A, B, C, D, E, H, and L), and sia (A and D) (Fig. 1A) (10–12), and no biofilm biosynthesis genes were found in the HLMP strain (Fig. 1B). Genome sequencing offers comparative insights into genomic arrangements focusing on genes associated with biofilm formation. Genomic maps were generated by Proksee (13).

**TABLE 1:** Summary of final draft assembly and annotation statistics

Metrics	HLHR	HLMP
Genome size (Mb)	6.4	5.2
*N*_50_ contig (kb)	424.7	465.7
Total reads	4,846,575	3,907,458
Total DNA sequenced (gigabase)	1.5	1.2
GC (%)	64.12	63.17
Protein coding genes	5,818	4,704
Completeness (%)	97.88	99.13
Number of contigs	97	38
Number of scaffolds	87	23

All bioinformatics tools were used with default parameters unless there were specific requirements.

## Data Availability

The genome sequence draft of *Pseudomonas aeruginosa* strain HLHR and *Pseudomonas* sp. HLMP can be found in the National Center for Biotechnology Information (NCBI). Genome Sequence data have been deposited at GenBank under accessions JBDLLS000000000 and JBDLLR000000000 for *Pseudomonas aeruginosa* strain HLHR and *Pseudomonas* sp. HLMP, respectively. For *Pseudomonas aeruginosa* strain HLHR, the BioProject accession and SRA numbers are PRJNA1111405 and SRR32120367; for *Pseudomonas* sp. HLMP, the BioProject accession and SRA numbers are PRJNA1111369 and SRR32109358.
